# The North of England Obstetrical and Gynecological Society

**Published:** 1890-03

**Authors:** 


### THE NORTH OF ENGLAND OBSTETRICAL AND GYNECOLOGICAL SOCIETY.

THE adjourned meeting to complete the arrangements for the foundation of this Society was held at the Queen's Hotel, Manchester, on January 17th. Dr. Wallace, Liverpool, occupied the chair. After adopting the constitution and rules prepared by a provisional committee, the meeting proceeded to elect office-bearers for the first year. Dr. Wallace was elected President, and Dr. Lloyd Roberts, Manchester, Dr. McGowan, Oldham, Dr. Burton and Mr. William Alexander, Liverpool, Dr. Braithwaite and Mr. C. J. Wright, Leeds, Vice-Presidents. Dr. Wm. Walter, Manchester, was appointed Treasurer, Dr. Sinclair, Manchester, General Secretary, and Dr. Donald, Dr. H. Briggs, and Mr. Sydney Rumboll, Local Secretaries for Manchester, Liverpool, and Leeds respectively. In addition to these, a council of twenty-four members was formed, comprising eight representatives of each center, viz. :—For Manchester, Dr. Samuel Buckley, Dr. John Scott, Dr. Wm. Lauder, and Dr. Nesfield, Mr. J. H. Martin, Blackburn, Dr. Milne, Accrington, Dr. Robertson, Oldham, and Dr. Starkey Smith, Warrington; for Liverpool: Drs. J. Armstrong, T. B. Grimsdale, and William McFie-Campbell, Liverpool, T. S. Floyd and Hugh Miller, Birkenhead, Dr. Moore, Preston, Dr. Barron, Southport, and Dr. Alexander Hamilton, Chester; for Leeds: Mr. Mayo Robson, Dr. Hellier, and Mr. Edmund Robinson, Leeds, Dr. Hime, Bradford, Dr. Johnstone, Ilkley, Dr. Arthur Jackson, Sheffield, and Mr. J. F. Horne, Barnsley.

It was announced that the first meeting would be held at Manchester on the third Friday in February, when Dr. Wallace would read a paper on The Etiology, Prognosis, and Treatment of Localised Peritonitis.—*Medical Chronicle.*

